# Reviewed article: Tiroli CF, Scholze AR, Magnabosco GT, Barreto MFC, Bolorino N, Zandomenighi RC, Pieri FM. Risk factors associated with hepatitis C virus infection: population-based cross-sectional study, Paraná, 2007-2022. Epidemiol Serv Saude. 2026:35;e20250196

**DOI:** 10.1590/S2237-96222026v35e20250196.c

**Published:** 2026-04-10

**Authors:** Mariângela Ribeiro

**Affiliations:** 1 Universidade Estadual de Campinas, Campinas, SP, Brazil Universidade Estadual de Campinas Campinas SP Brazil

## First round comments

O terma é relevante e dentro do escopo da RESS

O título pode ser aprimorado: substituir 'anticorpo específico contra vírus da hepatite C' por infecção pelo vírus da hepatite C.

O manuscrito necessita de ampla revisão sobretudo na seção de métodos, além de discussão mais aprofundada das limitações do estudo.

A seção de métodos deve ser ajustada e seguir de forma sistemática o check list strobe para estudos observacionais: Delineamento do estudo; Contexto; Participantes; Variáveis (uniformizar as variáveis e categorias ao longo do manuscrito); Fontes de dados/ mensuração; Viés; Tamanho do estudo; Métodos estatísticos. (https://www.strobe-statement.org/).

Recomenda-se o aprimoramento dos métodos estatísticos conforme assinalado pelos revisores.

Recommendation: Major revision

## Second round comments

Prezados autores

Agradecemos o envio do manuscrito trata-se de tema relevante e pertinente ao conhecimento da epidemiologia das hepatites virais no estado do Paraná.

Foram feitas as modificações sugeridas pelos revisores.

Sugiro uma pequena modificação relativa ao uso de agonistas parciais de receptores opioides: na discussão é citado um princípio ativo específico (buprenorfina), sugiro retirá-lo e colocar de forma genérica "agonistas parciais de receptores opioides".

Após esta mínima adequação, recomendo a publicação do manuscrito.

Recommendation: Accept

